# A multifunctional Janus-inspired therapeutic patch enabling intraoperative control of tumor dissemination and unidirectional drug delivery

**DOI:** 10.1016/j.mtbio.2026.103478

**Published:** 2026-07-24

**Authors:** Xuan Pan, Xiaoming Wang, Qin Dang, Longjiang Chen, Guannan Wang, Xu Wang, Shihang Xi, Huaqing Zhang, Xiaosan Fang

**Affiliations:** a Department of Hepato-Biliary-Pancreatic Surgery, The First Affiliated Hospital of Wannan Medical University (Yijishan Hospital of Wannan Medical University), Wuhu, 241000, China; b Department of Pancreatic Surgery, Fudan University Shanghai Cancer Center, Shanghai, 200032, China; c State Key Laboratory of Natural Medicines, Department of Pharmaceutics, China Pharmaceutical University, Nanjing, 211198, China

**Keywords:** Implantable membrane, Unidirectional drug release, CTC capture, PDAC therapy, Postoperative recurrence

## Abstract

Postoperative recurrence and metastasis remain formidable challenges in pancreatic ductal adenocarcinoma (PDAC), primarily due to bleeding-driven tumor dissemination and residual microscopic lesions after resection. However, the current surgical operation offers limited intraoperative treatment strategies to prevent these events. Herein, a multifunctional Janus-inspired therapeutic patch (JTP) is designed to bridge surgical resection and localized chemotherapy within a single intraoperative implant. The JTP features a trilayer Janus architecture that enables integrated control over hemostasis, tumor interception, and intratumoral chemotherapy. Specifically, the inner gelatin-hyaluronic acid sponge enables rapid hemostasis (1.24 min) and capture of circulating tumor cells. The middle gemcitabine-loaded polylactic acid nanofiber layer enables sustained and local drug release of gemcitabine (GEM) toward the surgical resection site, while the outer hydrophobic polycaprolactone backing serves as a protective barrier, preventing peritoneal adhesion and drug diffusion in unwanted directions (less than 5%). In an orthotopic PDAC resection model, a single JTP implantation achieved 78% blood loss reduction, ∼89% tumor suppression, and substantial inhibition of hepatic metastasis, extending survival to 38 days with 80% survival rate. By transforming the surgical site into an active therapeutic interface, this Janus-inspired patch establishes an effective strategy that bridges surgery and intratumoral chemotherapy for effective postoperative PDAC control.

## Graphical abstract

**Figure:**
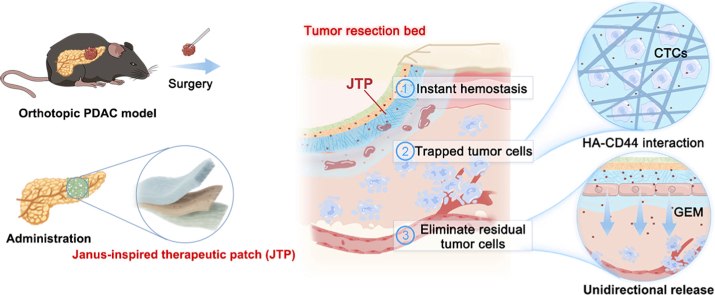

## 1 Introduction

Pancreatic ductal adenocarcinoma (PDAC) is one of the most lethal malignant tumors, characterized by high aggressiveness, early micrometastasis, and poor response to current therapies, with a 5-year survival rate below 13% [1,2]. Although surgical resection remains the primary curative approach, more than 50% of patients suffer from local relapse or distant metastasis within 2 years after surgery [3,4]. Such a high recurrence rate and metastasis rate are mainly attributed to the residual tiny tumor cells and tumor proliferation caused by bleeding during resection. The intraoperative period thus represents a critical therapeutic window, during which timely intervention can directly target residual tumor foci and interrupt bleeding-induced tumor dissemination, bridging the treatment gap between surgery and postoperative therapy [5,6].

Postoperative recurrence is mainly caused by tiny tumor residues that infiltrate the pancreatic bed plexus and lymphatic vessels [7]. Although intraoperative radiotherapy is an existing clinical treatment plan to inhibit postoperative recurrence and metastasis, it is not only ineffective but also limited by the interdisciplinary cooperation of professional teams and strict patient selection standards [8,9]. Moreover, surgical trauma-induced hemorrhage promotes tumor cell intravasation into circulation, elevating circulating tumor cell (CTC) burdens and amplifying risks for both locoregional recurrence and distant metastatic spread [10,11]. This bleeding-driven dissemination transforms the surgical field from a curative site into a potential source of recurrence and distant metastasis. Worse still, intraoperative bleeding will aggravate the risk of surgery, increase complications, delay subsequent adjuvant therapy, and further promote the recurrence and metastasis of PDAC [12,13]. Hence, an integrated strategy capable of immediate hemostasis, CTCs sequestration, and localized chemotherapy is urgently needed.

Conventional adjuvant therapies, including systemic chemotherapy and localized chemoradiotherapy, rarely eradicate circulating or residual tumor cells due to tumor heterogeneity, adaptive drug resistance, and stromal shielding [[14], [15], [16], [17]]. Likewise, commonly used hemostatic agents provide only passive physical occlusion, providing limited efficacy in diffuse bleeding and no capacity to intercept bleeding-driven CTCs dissemination. To overcome these challenges, localized drug delivery systems and multifunctional hemostatic biomaterials have been actively explored as intraoperative adjuncts [18,19]. Hydrogels [20,21], nanofiber sponges [22,23], and mesoporous silica nanoparticles [24,25] have shown potential in localized drug release, wound sealing, and hemostatic control [26,27]. However, current systems still fail to achieve spatiotemporally controlled drug release and rapid hemostasis to prevent bleeding-induced tumor dissemination, and sufficient biosafety for intraoperative application.

**Fig. 1:**
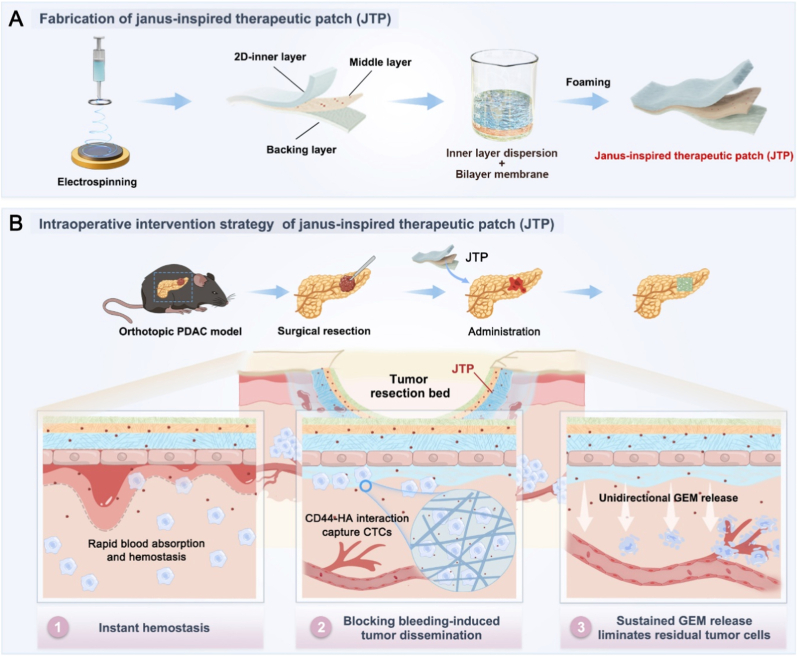
Schematic illustration of the synthesis and therapeutic mechanisms of multifunctional Janus-inspired therapeutic membrane (JTP) for preventing postoperative recurrence and metastasis in PDAC. (A) Stepwise fabrication of JTP via electrospinning combined with 3D foaming technology. (B) Multifunctional design of JTP combining hemostasis, tumor interception, unidirectional GEM delivery to bridge surgery and localized chemotherapy for postoperative PDAC treatment.

Here, we provide a multifunctional Janus-inspired therapeutic patch (JTP) as a conceptually integrated intraoperative platform that unites hemostasis, prevention of bleeding-induced tumor dissemination, and intratumoral chemotherapy within a single implant (Fig. 1A). Structurally, the JTP comprises three layers: (i) Inner layer: a gelatin/hyaluronic acid (HA) sponge that rapidly absorbs blood and binds CD44 receptors overexpressed on tumor cells to capture CTCs, (ii) Middle layer: a gemcitabine-loaded polylactic acid (PLA) fiber for unidirectional drug release to eliminate residual tumor cells and captured CTCs, and (iii) Backing layer: a polycaprolactone (PCL) backing that isolates the peritoneal cavity and minimizes off-target drug diffusion. Although the construct adopts a multilayer (sandwich-like) configuration, it is defined here as a therapeutic patch due to its functional asymmetry, with distinct roles assigned to the tissue-facing and outer surfaces. By engineering unidirectional therapeutic release and local microenvironmental modulation at the resection interface, this design transforms the surgical wound from a passive trauma site into an active therapeutic niche (Fig. 1B). This intraoperative patch provides a clinically relevant intraoperative intervention strategy for bridging surgery and localized chemotherapy, offering new translational potential for postoperative PDAC management.

## 2 Materials and methods

### 2.1 Materials

Gelatin (type B) was purchased from Sinopharm Chemical Reagent Co., Ltd. (China). Hyaluronic acid (HA, Mw = 2400 kDa) was obtained from Shandong Galaxy Bio-Tech Co., Ltd. (Shandong, China). Poly (D, L-lactide) (PLA, Viatel™ DL09E) was obtained from Ashland (Ireland). Gemcitabine (GEM) was purchased from Macklin (Shanghai, China). Formic acid (FA) and hexafluoroisopropanol (HFIP) were purchased from Aladdin (Shanghai, China).

### 2.2 Cells

NIH/3T3 cells (SCSP-646S) were procured from the Chinese Academy of Sciences (Shanghai, China). Pan02-Luc cells (IML-045) and Pan02 cells (IM-M092) were bought from Yimo Biotechnology Co., Ltd. (Xiamen, China). Pan02-GFP cells (GZQ0042) were bought from Shanghai Zhong Qiao Xin Zhou Biotechnology Co., Ltd (Shanghai, China). All cells were cultured under the rigorously controlled conditions, specifically at 37°C, 5% CO_2_, and 90% humidity. The culture medium was composed of Dulbecco's Modified Eagle Medium (DMEM; Gibco, USA), fetal bovine serum (FBS; Gibco, USA) at a volume ratio of 10%, and penicillin-streptomycin (Gibco, USA) at a volume ratio of 1%. The medium was changed every 24 h. Cell identification was performed through short tandem repeat (STR) typing and morphological observation.

### 2.3 Animals

Male C57BL/6 mice (6-8 weeks, 20-22 g) were purchased from Qinglongshan Animal Center (Jiangsu, China) and housed in an SPF animal facility. The animals were maintained on a standard diet and 45-55% relative humidity with a 12-h light-dark cycle. China Pharmaceutical University Ethics Committee approved the protocol for all animal experiments and ensured compliance with the Guide for Care and Use of Laboratory Animals.

### 2.4 Preparation of the JTP patch

PLA (400 mg) and GEM (400 mg) were dissolved in a solvent mixture (HFIP: DMF = 9:1, v/v) to prepare a mixed drug-loaded electrospinning solution with both the PLA and GEM at concentrations of 20 w/v%. PCL (200 mg) was dissolved in HFIP to prepare a concentration of 15 w/v% electrospinning solution. The drug-loaded electrospinning solution was transferred into a 5 mL syringe fitted with a 20 G needle. The receiving distance between the needle and the collector was set to 18 cm, the applied voltage to 9 kV, and the flow rate to 0.1 mm/min. PLA without GEM was processed under identical conditions to obtain the blank PLA membrane. Then, bilayer membrane was prepared by electrospinning the PCL solution onto GEM-loaded middle membrane for 1 h (20 G needle, 18 cm, 9 kV, 0.15 mm/min). A blank bilayer membrane fiber was prepared similarly using the blank PCL backing layer.

Gelatin (1 g) and HA (25 mg) were dissolved in a 5 mL solvent system (HFIP: FA = 7:3, v/v) to yield the 20 w/v% gelatin-HA electrospinning solution. The solution (4 mL) was transferred into a 5 mL syringe fitted with a 20 G needle, with the receiving distance set to 18 cm, the voltage to 15 kV and the flow rate to 0.2 mm/min. The thickness of the resulting gelatin-HA fiber was prepared in a range of 0.5-1 cm. After electrospinning, the collected fibers (50 mg) were foamed by liquid nitrogen to obtain JTP. The gelatin-JTP could be achieved by replacing gelatin-HA fiber with gelatin fiber without HA. If the gelatin-HA electrospinning solution was directly spun on the surface of bilayer membrane without 3D foaming, 2D-JTP could be prepared in identical conditions (20 G needle, 18 cm, 15 kV, 0.2 mm/min).

### 2.5 Morphology analysis

The morphology of the inner layer (gelatin-HA sponge) in the JTP was examined by scanning electron microscopy (SEM) (SU8020, Hitachi, Japan). Briefly, the inner layer was immersed in liquid nitrogen for 60 s and then fractured to expose the cross-section. The cross-section was then sprayed with gold and mounted on the sample stage with conductive adhesive for observing and imaging. The morphology of the drug-loaded middle layer, blank middle layer and backing layer were also observed by SEM. The porosity of the samples was quantitatively analyzed using ImageJ software and calculated using the following formula:

(1) $$\text{Porosity}\;\left(\%\right)=\frac{\text{pore}\;\text{area}}{\text{total}\;\text{area}}\times 100\%$$

### 2.6 Water contact angle measurement

Inner layer, middle layer and backing layer were prepared separately. The water contact angles were measured with a contact angle measuring instrument (OCA 50, Dataphysics, Germany) to assess the hydrophobicity of each layer. 2 μL of deionized water was deposited on the surface of the sample, and the morphology of water droplets was captured after stabilization for 60 s. The water contact angle was calculated using the Young-Laplace equation. The contact angles of three regions on each layer were measured, and the average value was taken.

### 2.7 In vitro drug release

Phosphate-buffered saline (PBS) was chosen as the release medium to study the drug release profile in vitro. 10 mg/mL GEM standard solution was prepared and then serially diluted to 1, 5, 10, 25, 50, and 100 μg/mL, which were sequentially analyzed by a high-performance liquid chromatography system (HPLC, 1260 Infinity II, Agilent, USA), and the corresponding peak areas were recorded. Peak area was linearly correlated with the concentration of GEM solution to establish a standard curve for further quantification. JTP containing 2 mg GEM (M_total_) was weighed and dissolved in PBS (V_total_ = 20 mL) and gently shaken at 100 rpm at 37°C. At 0, 2, 4, 8, 12, 24, 36, 48, 72, 120, 168, 216, 276, and 336 h, 0.5 mL (V_0_) of release medium was collected. The peak and retention time of the collected samples were determined. The peak areas were measured, and the corresponding concentrations (C_n_) were then calculated.

(2) $$\text{Cumulative}\;\text{GEM}\;\text{release}\;\left(\%\right)=\frac{C_{n}{\cdot V}_{\text{total}}+\sum_{i=1}^{n‐1}C_{i}{\cdot V}_{0}}{M_{\text{total}}}\times 100\%$$

### 2.8 In vitro unidirectional drug release

Unidirectional drug release behavior of JTP was studied using Transwell plates. JTP loading 1 mg GEM was cut into discs and put in the Transwell chamber, with backing layer face up and inner layer side down. Bilayer sponge was cut into same size and put in the Transwell plates with middle layer face up and inner layer side down. The middle layer of the same size was chosen to be the control. 400 μL PBS (pH = 7.4) was added to the dish, and 400 μL PBS was added to the inner chamber. After immersion for 12 h, PBS (100 μL) from the inner and outer chambers was extracted, respectively. The relative and absolute concentration of GEM was determined to evaluate the drug release behavior.

### 2.9 In vitro degradation

JTP were weighed (W_0_) and immersed in 10 mL PBS (pH 7.4) at 37°C in a thermostatic oscillator (SHKE6000-1CE, Thermo, USA) to simulate physiological conditions. At predetermined intervals (0, 1, 3, 6, 12, 18 d), the samples were removed, washed gently with distilled water, and lyophilized for 24 h. The dried samples were then weighed carefully (W_n_). After weighing, the samples were then transferred back to 10 mL fresh medium and incubated under the identical conditions till the next time point. The weight residual (%) was calculated as:

(3) $$\text{Weight}\;\text{residual}\;\left(\%\right)=\frac{W_{n}}{W_{0}}\times 100\%$$

### 2.10 In vitro hemostasis capability assessment

To qualitatively evaluate the hemostatic performance of the materials, Gelatin sponge and JTP were cut into identical volumes and placed in petri dishes. Then, EDTA-K2 containing whole blood (100 μL) was dropped onto each sample. Whole blood of the same volume was dropped directly onto the dish as the control group. All dishes were incubated at 37°C for 5 min, after which 50 mL of distilled water was gently added. The hemostasis degree in each group was observed and recorded.

To quantitatively assess the hemostatic capability, JTP and commercial gelatin sponge (Sponge) were placed in beakers separately. 100 μL EDTA-K2-containing whole blood was slowly added to the surface of each material and incubated at 37°C for 5 min in a thermostatic oscillator. Subsequently, distilled water (50 mL) was gently added into the beaker with the clots not being disturbed. The absorbance of the diluted blood samples at 540 nm was measured by an ultraviolet-visible spectrometer (UV-2450 PC, Shimadzu, Kyoto, Japan) to evaluate the hemostatic capacity of the patch. Assuming that the absorbance of 100 μL whole blood in 50 mL distilled water equals 100%, the blood-clotting index (BCI) of different samples could be calculated as:

(4) $$\text{BCI}=\frac{{\text{Abs}}_{540\;\text{nm}}\;\text{of}\;\text{sample}‐\text{treated}\;\text{blood}}{{\text{Abs}}_{540\;\text{nm}}\;\text{of}\;\text{blood}\;\text{in}\;\text{water}}\times 100\%$$

Sponge and JTP were cut into cubes with the same volume (1 cm × 1 cm × 0.5 cm) (V_dry_) and weighed respectively (W_dry_). The dry samples were then immersed in 5 mL saline or EDTA-K2-containing whole blood. After 30 min, the samples were blotted on filter paper to remove the fluid on the surface, and were immediately weighed to determine the weight (W_wet_). The fluid absorption ratio was calculated as:

(5) $$\text{Fluid}\;\text{absorption}\;\text{ratio}=\frac{W_{\text{wet}}\;‐\;W_{\text{dry}}}{V_{\text{dry}}}\times 100\%$$

### 2.11 In vivo hemostasis capability assessment

Nine healthy C57BL/6 mice were randomly divided into three groups which were labeled as the control group, gelatin sponge group, and JTP group respectively with three mice in each group. After the mice were completely anesthetized by 400 μL 1.25% tribromoethanol (M2920, AibeiBio, Nanjing), the skin along the midline of the abdomen was incised to expose the liver. The liver was placed on a dry filter paper to observe and record the bleeding situation. Then, scratch wounds were made on the surface of the liver to induce liver bleeding. After the scratches were made, gelatin sponge group and JTP group mice were immediately treated with the corresponding materials on the wounds considering mice with no treatment as the control group. Subsequently, the bleeding time and blood loss of each mouse were recorded.

### 2.12 Construction of postoperative PDAC model

To establish a postoperative PDAC model, male C57BL/6 mice were anesthetized and a 1.0 cm incision was made to expose the tail of the pancreas. Then, 50 μL of DMEM/Matrigel basement gel (1:1, v/v) containing approximately 1 × 10^6^ Pan02-Luc cells was slowly injected into the pancreatic tail. The abdominal wall and skin wounds were then sutured. On the 21st day after the surgery, when the fluorescence intensity of the tumor exceeded 1 × 10^7^ p/s/cm^2^/sr, the mice were anesthetized again and underwent a second laparotomy to expose the tumor site. The spleen vessels were ligated and part of the tumor at the pancreatic tail was removed. Approximately 10% of the tumor tissue was retained to simulate the residual micro-tumors after the surgery.

### 2.13 In vivo spatial distribution of JTP

50 μL of DiR fluorescent dye (10 μg/μL) was mixed with 1 ml of 20% polylactic acid (PLA) spinning solution to prepare a ^DiR^JTP patch. PDAC-bearing mice were randomly divided into two groups (n = 3): (1) Free DiR in situ administration group; (2) ^DiR^JTP patch group. *In vivo* fluorescence imaging was performed at 1 h, 3 h, 24 h, 48 h, and 72 h after administration via a small animal imaging system (VISQUE InVivo Smart-LF, Vieworks Co., Ltd., Korea). The heart, liver, spleen, lung, kidney, and tumor were collected after the mice were sacrificed at predetermined time intervals for ex vivo fluorescence imaging. The distribution and retention of the patch in the target tissues and major organs were quantitatively analyzed based on fluorescence intensity.

Tumor tissues were harvested at 6 h, 1 d, 7 d, and 14 d after JTP implantation and divided into peripheral and core regions based on the distance from the implantation surface. The outer 3-4 mm region was defined as the tumor periphery, while the inner 1-2 mm region was defined as the tumor core. Adjacent abdominal wall tissues and blood samples were also collected. All tissue samples were weighed, homogenized, and processed for GEM extraction. GEM concentrations were quantified using HPLC.

### 2.14 In vitro cell compatibility tests

The blank JTP, inner layer, and backing layer were cut into shapes consistent with the size of 24-well plates to ensure the bottom of the well was completely covered. After the cut patch was exposed to ultraviolet light for 1 h, sterilized patch was then placed on the bottom of the 24-well plates using sterile forceps. NIH/3T3 cells were plated into 24-well plates at a density of 1 × 10^5^ cells/well. After incubation for 24 h, MTT reagent was added for 4 h, followed by replacement of DMSO to dissolve the resulting formazan crystals. The 24-well plates were then shaken at 100 rpm for 15 min at 37°C to achieve complete dissolution and uniform mixing of the formazan. The absorbance of each well at 570 nm was measured using a microplate reader (Varioskan LUX, Thermo Fisher Scientific, Inc., MA, USA), and cell viability was calculated using the following formula:

(6) $$\text{Cell}\;\text{viability}\;\left(\%\right)=\frac{{O.D.}_{\text{sample}}}{{O.D.}_{\text{control}}}\times 100\%$$

### 2.15 Western blot

Pan02 and NIH/3T3 cells were cultured and lysed on ice for 30 min. Then the lysates were centrifuged and the total protein concentration in the supernatant was quantified via a BCA assay. Equivalent amounts of protein (25 μg) were mixed with loading buffer, denatured, resolved by SDS-PAGE, and subsequently transferred onto PVDF membranes. After blocking with 5% non-fat milk for 1 h at room temperature, the membranes were incubated overnight at 4°C with a primary antibody against CD44 (1:2000, ab157107, Abcam). Following TBST washes, the membranes were treated with HRP-conjugated secondary antibodies for 1 h, washed twice, and visualized using an enhanced chemiluminescence (ECL) system. The resulting bands were imaged and quantified, with β-actin (1: 5000, 200068-8F10, ZENBIO) serving as the internal reference.

### 2.16 Evaluation of tumor capture ability in vitro

First, NIH/3T3 cells and Pan02 cells were stained with Hoechst 33258 staining solution (C1018, Beyotime Biotechnology Co., Ltd., Shanghai) for 15 min. Following the staining procedure, the cells were washed twice with PBS buffer to ensure the removal of all unbound dyeing agent. The blank bilayer membrane, 2D-JTP, blank gelatin-JTP, and blank JTP samples were tailored to the size of the 24-well plates and underwent 1 h of ultraviolet illumination. The sterilized samples were subsequently positioned flat at the base of the 24-well plates. The stained NIH/3T3 cell suspension and Pan02 cell suspension were added to the prepared wells, with approximately 1 × 10^5^ cells/well. The 24-well plates were incubated in the cell culture incubator for 1 h. Then, it was gently washed twice with PBS to remove the unbound cells. The spatial distribution of cells in each well was meticulously observed using an inverted microscope (Olympus FV3000 microscope, Japan). Several random visual areas were selected for the purpose of counting the captured cells. Then, the total number of captured cells was calculated by amplifying the proportion of the field area to the entire well area. The efficiency of cell capture was defined as the following formula:

(7) $$\text{Capture}\;\text{efficiency}=\frac{\text{number}\;\text{of}\;\text{captured}\;\text{cells}}{\text{number}\;\text{of}\;\text{initially}\;\text{introduced}\;\text{cells}}\times 100\%$$

### 2.17 In vivo capture

PDAC tumor-bearing mice were anesthetized, and PDAC tumors were surgically resected. Then Pan02-Luc cells (1 × 10^5^, 100 μL) were subcutaneous injected to the bleeding site. blank bilayer membrane, blank 2D-JTP, blank gelatin-JTP, and blank JTP (1 cm × 1 cm) were immediately implanted on the bleeding site. After fixed time intervals, the implants were taken out and imaged by a small animal imaging system with automatic ROI quantitative analysis.

### 2.18 MTT assay

JTP patch was cut into small pieces (0.5 g) and irradiated with ultraviolet light for 1 h. Every piece was transferred to 1 mL of DMEM medium, then cultured at 100 rpm and 37°C for 24 h. The drug concentration in the extracted solution was detected by ultraviolet light. The patches were diluted to 0.001 μM, 0.1 μM, 0.5 μM, 1 μM, 10 μM, 50 μM, 100 μM, 125 μM, 200 μM, 500 μM, and 1000 μM with DMEM. Free GEM solution at the same concentration was prepared by directly dissolving GEM powder in DMEM. All the prepared solutions described above were carefully filtered through a 0.22 μm filter membrane (RSF33VQ2B, Jiangsu Green Union Science Instrument Co., Ltd.) to obtain a sterile extraction solution. The negative control group was assembled using the same volume of blank DMEM, with 6 replicates established for each group. Subsequently, Pan02 was incubated in the 96-well plate at a density of 10^4^ per well for 24 h incubation, followed by 10 μL of 5 mg/mL MTT solution being added to each well and co-cultured for 4 h. After 4 h incubation, the MTT solution was replaced by 150 μL DMSO. The 96-well plate was placed in a constant temperature shaker at 100 rpm for 15 min to fully dissolve and mix the formazan. The absorbance value at 570 nm was measured using a microplate reader to calculate the cell viability, and the IC50 value of each group was calculated.

### 2.19 Live/dead cell staining assay

Pan02 cells were seeded in 24-well plates and cultured until reaching approximately 90% confluence before subsequent treatments. Then, after the 24 h incubation with blank DMEM, blank JTP, free GEM, and JTP, cells were gently washed twice with PBS and subjected to live/dead staining (Abcam, ab287858). Briefly, the cells were incubated with a staining solution containing Calcein-AM and propidium iodide (PI) at 37°C for 20-30 min in the dark. Following washing with PBS, fluorescence images were immediately captured using an inverted fluorescence microscope. Live cells exhibited green fluorescence due to intracellular esterase-mediated conversion of Calcein-AM to Calcein, whereas dead cells with compromised membrane integrity were stained red by PI. Quantitative analysis of fluorescence images was performed using ImageJ software to determine the ratio of live to dead cells for both drug-loaded and non-loaded groups, allowing assessment of the effect of the treatment on cell viability.

### 2.20 Cell apoptosis evaluation

The blank JTP, GEM, and JTP patch were diced into small pieces and subjected to ultraviolet irradiation for 1 h. Every patch (0.5 g) was then cut and added to 1 mL of DMEM medium, followed by incubation at 100 rpm and 37°C for 24 h. The drug concentration in the extraction solution was detected by ultraviolet light, and the patch was diluted to 100 μM using DMEM. Pan02 cells were seeded in 96-well plates at a density of 2 × 10^4^ per well and incubated overnight in the cell culture incubator. The used culture medium was discarded, and 100 μL of the following extraction solutions were added to each group: serum-free DMEM medium, blank JTP, GEM, and JTP extraction solutions. The equivalent volume of serum-free DMEM was used as the negative control group, and each group was set up with 3 replicates. Subsequently, the 96-well plate was returned to the incubator for 24 h. After that, cells were washed twice with PBS to eliminate any remaining culture medium and extraction solutions. After washing, the cells were suspended in binding buffer. Annexin V and PI (5 μL/group; C1062M, Shanghai Beyotime Biotechnology Co., Ltd.) were introduced into the cell suspension, mixed gently, and incubated in the dark at room temperature for 15 min. The cells were detected by flow cytometry (BD FACSCalibur, USA) within 1 h. The fluorescence signals of Annexin V and PI were analyzed to distinguish between live cells, early apoptotic cells, late apoptotic cells, and necrotic cells.

### 2.21 Safety evaluation

Healthy C57BL/6 mice were randomly divided into three groups (n = 3): The treatments included intravenous saline (control group), intravenous GEM (GEM i.v. group), and multifunctional Janus-inspired membrane implantation (JTP) (1 cm × 1 cm), respectively. At designated time points after administration, 500 μL of mouse blood was collected into heparin sodium tubes. White blood cell count (WBC), platelet count (PLT), lymphocyte count (LYMPH), and red blood cell count (RBC) were determined with an automatic hematology analyzer (Mindray BC-6000). Lactate dehydrogenase (LDH; cardiac function parameters), creatine kinase (CK; cardiac function parameters), alkaline phosphatase (AKP; hepatic function parameters), alanine transaminase (ALT; hepatic function parameters), blood urea nitrogen (BUN; renal function parameters) and creatinine (CRE; renal function parameters) were detected with an automated chemistry analyzer (Polaris c2000). Major organs (heart, liver, spleen, lung, and kidney) were harvested, fixed, and processed for histological examination with H&E staining.

### 2.22 In vivo antitumor efficacy

The postoperative PDAC model was established, as mentioned above. The postoperative mice were randomly divided into five groups (n = 8): The treatments included intravenous saline (control group), intravenous GEM (GEM i.v. group), drug-free membrane implantation (blank JTP group), the bilayer membrane integrating GEM-loaded middle layer and backing layer, and multifunctional Janus-inspired membrane implantation (JTP) (1 cm × 1 cm), respectively. Photographs were taken to record the key surgical steps. The fluorescence intensity at the tumor site was monitored daily with a small animal imaging system, and the automatic quantitative analysis of the region of interest (ROI) is conducted. The tumor recurrence of the mice was monitored every day. The time of tumor recurrence was determined when the fluorescence intensity exceeded 1 × 10^7^ p/s/cm^2^/sr. The survival time of mice was tracked for 40 days post-treatment (n = 5). The survival rate was obtained and the Kaplan-Meier curve was plotted. All the surviving mice were uniformly sacrificed after the postoperative survival period assessment was completed. On the 15th day after surgery, the mice were sacrificed (n = 3), and the tumor tissues were isolated and weighed. Tumor tissues were collected, fixed with 4% paraformaldehyde, dehydrated through graded ethanol, cleared with xylene and embedded in paraffin. Paraffin blocks were sectioned into 4-5 μm serial slices, mounted on glass slides, dewaxed in xylene, rehydrated through graded ethanol, and stained for histological analysis including H&E staining, TUNEL assay (GDP1042, Servicebio) for apoptosis, Ki67 immunostaining (1:1000, PA5-19462, ThermoFisher) and PCNA (1:1000, 13-3900, ThermoFisher) for cell proliferation.

### 2.23 Quantification of circulating tumor cells in peripheral blood

Peripheral blood samples were collected from mice at 6 h, 1 d, 7 d, and 14 d after surgery and JTP implantation. Approximately 200 μL of blood was obtained from the orbital sinus and collected into anticoagulant-containing tubes. Red blood cells were lysed using erythrocyte lysis buffer, and the remaining nucleated cells were collected by centrifugation. CTCs were identified based on GFP fluorescence derived from the GFP-labeled Pan02 orthotopic tumor model. The isolated cells were resuspended in PBS and analyzed under a fluorescence microscope, and GFP-positive cells were counted as CTCs. The number of CTCs was normalized to blood volume and expressed as cells/mL of blood. Mice treated with saline served as the control group.

### 2.24 Calcein-AM/PI staining of CTCs on JTP

Captured tumor cells on the JTP surface were evaluated using Calcein-AM/PI live/dead staining at 12 h, 24 h, 48 h, and 72 h after cell seeding. At each time point, the samples were gently washed with PBS to remove non-adherent cells and incubated with Calcein-AM and propidium iodide (PI) staining solution according to the manufacturer's instructions. After incubation at 37°C for 30 min in the dark, the samples were washed with PBS and observed under a fluorescence microscope. Live cells were stained green by Calcein-AM, while dead cells were stained red by PI. Representative fluorescence images were acquired with a scale bar of 10 μm.

### 2.25 Characteristics of liver metastasis

After the end of the drug administration cycle, the PDAC-bearing mice were injected with sodium fluorescein and then sacrificed. The livers were excised and rinsed in PBS to remove surface blood and photographed to record macroscopic nodules. Then, livers were placed flat on a black background plate and hepatic fluorescence intensity was measured with a small animal imaging system. Tissue sections were prepared for H&E staining and the metastatic foci were observed under a microscope to determine the occurrence of metastasis and calculate the metastatic area ratio.

### 2.26 Statistical analysis

Statistical analyses were performed using SPSS. One-way analysis of variance (ANOVA) with Tukey's correction was used to analyze if the scores of multiple groups differ on a single variable. T test was used to analyze the statistical significance between the two groups. All data were presented as mean ± SD. Differences were considered statistically significant at a level of *P < 0.05, **P < 0.01, ***P < 0.001.

## 3 Results

### 3.1 Preparation and characterization of the implantable janus-inspired therapeutic patch

**Fig. 2:**
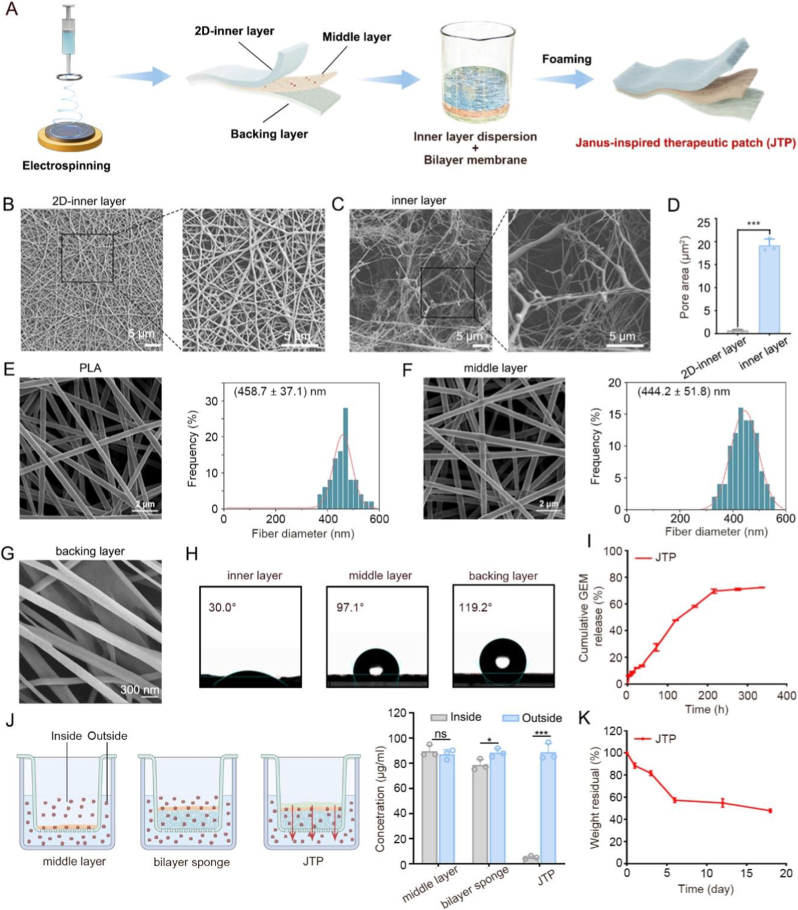
Preparation and characterization of JTP. (A) Schematic illustration of the preparation process of JTP via electrospinning and a 3D foaming process. SEM images of (B) 2D-inner layer and (C) inner layer (bar = 5 μm). (D) Pore areas of the 2D-inner layer and the inner layer. SEM images and fiber diameter distribution of (E) PLA and (F) middle layer (bar = 2 μm). (G) SEM image of PCL (bar = 300 nm). (H) Water contact angles of the inner layer, the middle layer, and the backing layer, respectively. (I) GEM release profiles of JTP for 14 days. (J) Schematic of GEM delivery behavior of the middle layer, bilayer sponge, and JTP in a Transwell model and the quantification of GEM concentration in inner/outer chambers. (K) Residual weight profile of JTP for 18 days. Data were expressed as mean ± SD (n = 3). *P < 0.05, ***P < 0.001, ns P> 0.05.

To enable intraoperative implantation with integrated unidirectional GEM delivery, hemostasis, and tumor cell interception, a multifunctional Janus-inspired therapeutic patch (JTP) was fabricated via the combination of electrospinning with a 3D foaming technique (Fig. 2A). The patch exhibited a trilayer Janus configuration designed to regulate postoperative drug distribution and surgical microenvironment in a spatiotemporally controlled manner.

Specifically, a GEM-loaded poly(lactic acid) (PLA) nanofiber membrane was fabricated through electrospinning to serve as the middle chemotherapeutic layer, enabling sustained and unidirectional GEM release. A hydrophobic polycaprolactone (PCL) film was subsequently deposited as a PCL backing layer, forming a bilayer membrane that prevents drug diffusion into the peritoneal cavity and minimizes postoperative adhesion. To endow the system with hemostatic and tumor-intercepting capability, a gelatin-hyaluronic acid (HA) fibrous network was converted into a 3D porous sponge through 3D foaming, yielding a highly absorbent and bioactive layer. This top hemostatic-capture layer was then integrated onto the bilayer membrane through freeze-drying, completing the Janus-inspired therapeutic patch (JTP) assembly. Through the morphological analysis of scanning electron microscopy (SEM), obvious structural changes in the preparation process were observed. As shown in Fig. 2B, gelatin-HA fibers (2D-inner layer) presented a dense and uniform fiber network with an average diameter of about 500 nm. After 3D foaming, the structure expanded into a loose and porous sponge-like structure, and the average hole area increased from 0.7 μm^2^ to 19.2 μm^2^ (Fig. 2C and D)**.**

This structural transition could markedly enhance water absorption and specific surface area, favoring rapid blood uptake and cell entrapment for achieving fast hemostasis and the interception of circulating tumor cells (CTCs). As shown in Fig. 2E, the drug-free PLA membrane exhibited an average fiber diameter of (458.7 ± 37.1) nm with a smooth surface, indicating excellent film-forming capability of PLA. In contrast, the GEM-loaded PLA nanofibers (middle layer) exhibited negligible morphological differences with a comparable average diameter of 444.2 ± 51.8 nm (Fig. 2F), indicating uniform GEM incorporation without compromising spinnability. And, the PCL backing layer presented a tightly packed fibrous structure (Fig. 2G), ensuring mechanical integrity and directional barrier function. Using Fourier transform infrared spectroscopy (FTIR) and X-ray diffraction (XRD) [28,29], the molecular state and intermolecular interaction of GEM in the membrane were further studied. The FTIR spectrum of JTP showed that GEM was successfully mixed into the membrane, and the characteristic peaks of PLA and GEM have been confirmed (Fig. S1, supplementary information). The slight weakening of GEM characteristic peaks (3410 cm^−1^ and 1658 cm^−1^) indicated that there was a potential intermolecular interaction between GEM and PLA nanofibers, such as hydrogen bonds or van der Waals forces. The XRD analysis results shown in Fig. S2 (Supplementary Information) further supported the above conclusion.

To further evaluate the functional characteristics of JTP, its layer-specific wettability, drug release behavior, directional transport properties, and degradation behavior were evaluated in detail. As illustrated in the water contact angle results (Fig. 2H), both the PLA layer (97.1°) and the PCL backing layer (119.2°) showed strong hydrophobicity, which was favorable for the sustained release of GEM and preventing the infiltration of surrounding tissues, thus minimizing drug leakage into the peritoneal cavity and reducing adhesion formation. In sharp contrast, the 3D-foamed inner layer displayed a markedly low contact angle of approximately 30°, which suggested excellent hydrophilicity that would guarantee an intimate contact with a bleeding surgical bed to accelerate hemostasis and improve inner layer-cell interactions. Then, we tested the GEM release profile and the unidirectional release behavior of the Janus-structured membrane.

As shown in Fig. 2I, JTP showed the continuous release of GEM, with a cumulative release of about 80%, and reached the platform period on the 9th day. This sustained drug release was due to the uniform distribution of GEM in PLA fibers. Subsequently, we used the extracorporeal Transwell model to evaluate the unidirectional GEM release capability provided by the Janus structure. As shown in Fig. 2J and Fig. S3 (Supplementary Information), the GEM-loaded PLA layer (middle layer), the bilayer sponge without PCL backing (bilayer sponge), and the Janus-inspired membrane group (JTP) were placed in the inner chamber, respectively. The results showed that similar GEM concentrations were detected in both chambers in the intermediate layer group, indicating that GEM was released in two directions. However, in the bilayer sponge group, the concentration of GEM in the lumen was much higher than that of the outer cavity, indicating that the partially released GEM was partially retained in the cavernous membrane, thus maintaining a local high concentration, which was enough to kill tumor cells. In contrast, in the JTP group, the GEM concentration of the outer cavity was 17.1 times that of the lumen, and the GEM concentration detected in the lumen was less than 5%, indicating a unidirectional release behavior. This drug release property endowed by the PCL backing layer allows JTP to enhance drug accumulation at the resection bed while preventing peritoneal adhesion and off-target toxicity.

As shown in Fig. 2K, the in vitro degradation of JTP presented a typical biphasic characteristic: the mass drops rapidly by about 40% in the first 5 days, and then entered a slow and more stable degradation stage. This degradation behavior reflected the degradation dynamics of the specific layers in the Janus structure. It is worth noting that due to the water absorption, the inner layer of gelatin/HA degrades rapidly, while the intermediate layer of the drug carrier degrades slowly, which is conducive to maintaining the continuous release of the drug. These results confirm that JTP has a clearly Janus structure, in which GEM is uniformly dispersed, degradation is controlled, and the drug release direction is consistent, thus ensuring stable structural integrity and continuous local chemotherapy drug delivery during postoperative implantation.

### 3.2 JTP promoted rapid in vitro clotting and efficient in vivo hemostasis

**Fig. 3:**
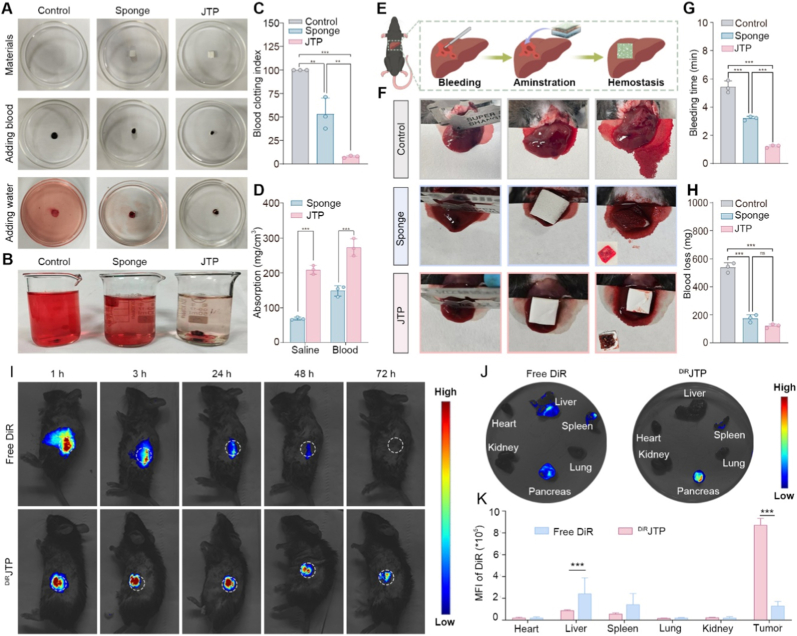
Hemostatic effect and delivery performance of JTP. (A) Photographs showing the blood coagulation process and (B) the status of the commercial sponge and JTP after clot formation. (C) Blood clotting index of various materials (n = 3). (D) Saline and blood absorption ratio of different samples. (E) Schematic depiction of the in vivo liver hemorrhage model establishment. (F) Representative images of mouse livers during the hemostatic procedure. The livers were incised and subsequently covered with a gelatin sponge or JTP. Scratched liver without treatment was used as control. (G) Bleeding time of different materials (n = 3). (H) Quantification of blood loss by measuring the weight of absorbed blood on filter paper (n = 3). (I, J) Representative fluorescence images showing both in vivo (white dashed circle indicates the tumor region) and ex vivo (heart, liver, spleen, lung, kidney, and tumor) biodistribution of model drug DiR in mice. (K) Mean fluorescence intensity (MFI) obtained from ex vivo images 24 h post-administration. Data were presented as mean ± S.D., **P < 0.01, ***P < 0.001, ns P > 0.05.

Due to its loose architecture and hydrophilicity, JTP facing the surgical bed enabled rapid blood absorption, while the collagen-mimicking activity of gelatin facilitated platelet activation and thrombus formation, thereby achieving efficient hemostasis. To preliminarily assess the clotting ability, whole blood containing EDTA-2Na and a commercially available gelatin sponge (Sponge) were used as controls. As shown in Fig. 3A and B, upon the addition of water, the JTP-treated samples exhibited negligible color change, revealing that JTP was capable of inducing effective blood coagulation within 5 min. In contrast, commercial gelatin sponge only coagulated a portion of the blood, resulting in a pale red rinsing solution. Meanwhile, the anticoagulant effect of EDTA-2Na completely inhibited thrombus formation in the control group; upon addition of distilled water, extensive hemolysis occurred, turning the solution deep red, indicating complete coagulation failure. As shown in Fig. 3C, the sponge showed a significantly higher blood coagulation index (BCI) of 53.36, which was 6.7 times higher than JTP (7.95). Because a higher BCI value corresponds to weaker coagulation ability, these results clearly demonstrate the superior clotting performance of JTP. Such enhancement can be attributed to the higher porosity and larger specific surface area of JTP, which promotes blood cell adsorption and fibrin network formation. Given the importance of blood absorption capacity for rapid coagulation, we further investigated the liquid absorption properties of different preparations. As shown in Fig. 3D, the blood absorption rate (w/v) of JTP was 1.84 times higher than that of the commercial sponge, indicating that its highly porous structure effectively facilitates blood uptake, platelet adhesion, and cellular aggregation. Collectively, these results demonstrate that JTP exhibits excellent in vitro hemostatic performance, providing a strong foundation for effective hemorrhage control during intraoperative application.

To further assess in vivo hemostatic efficacy of JTP, a mouse liver scratch model was established by creating a 1 cm incision on the hepatic surface. This model is a widely used, well-established, and highly reproducible model for assessing hemostatic biomaterials, providing stable bleeding and enabling quantitative comparison among different materials (Fig. 3E). As shown in Fig. 3F, the untreated group exhibited continuous bleeding, confirming that spontaneous hemostasis could not be achieved under this condition. After implantation, the sponge quickly absorbed blood, but its poor adhesion to the moist liver surface resulted in peripheral blood leakage, consistent with previous report [30]. In contrast, owing to its porous structure and hydrophilic functional groups, JTP quickly absorbed blood and underwent moderate deformation to closely conform to the irregular wound, thereby minimizing lateral leakage. Following removal, the wound site remained dry with fully coagulated blood in the sponge, visually confirming its hemostatic efficacy. Hemostasis time was recorded to quantitatively assess the efficacy of each treatment. As shown in Fig. 3G and H, in the absence of any hemostatic treatment, the liver lost 537.84 mg of blood in 5.45 min. After application of the commercial sponge, both the blood loss and hemostasis time markedly reduced to 173.98 mg and 3.23 min, respectively. In contrast, the wound treated with JTP achieved the fastest hemostasis (1.24 min) with 120.36 mg blood loss, which was only 22% of the untreated group and 69% of the commercial control. In general, the desirable hemostatic performance of JTP could be described as follows: when implanted at the postoperative pancreatic wound site, its loose porous and hydrophilic structure rapidly absorbed blood and retained cellular components within surface pores, facilitating platelet aggregation and coagulation. Meanwhile, the collagen-mimicking activity of gelatin further activated platelets to form stable thrombi, thereby achieving effective hemostasis.

### 3.3 JTP exhibited enhanced tumor cavity retention and sustained drug delivery performance

To visualize the biodistribution, retention, and sustained drug delivery performance of JTP within the tumor cavity, a near-infrared fluorescent probe DiR was used to prepare fluorescently labeled ^DiR^JTP. Then, a postoperative orthotopic PDAC model was established by inoculating Pan02 cells into the pancreas of C57BL/6 mice followed by surgical tumor resection. The postoperative PDAC model was evidenced by the bioluminescence signal (BLI) changes of Pan02-Luc cells before and after surgery (Fig. S4, Supporting Information), which indicated a resection efficiency of approximately 90%. To assess spatial GEM distribution in vivo, fluorescence-based tracking and HPLC analysis were performed. DiR was employed as a fluorescent surrogate for GEM, enabling real-time visualization of payload distribution. Immediately after tumor removal, ^DiR^JTP was implanted at the resection site. Compared with intratumoral injection of free DiR, ^DiR^JTP provided stronger and more localized fluorescence at the tumor site with minimal off-target distribution (Fig. 3I). In addition, intratumoral injection of free DiR group, although initially showing high fluorescence in tumor cavity, rapidly lost signal due to fast clearance and systemic metabolism, resulting in strong hepatic accumulation (Fig. 3J). In contrast, ^DiR^JTP maintained stable and prolonged intratumoral fluorescence, showing a 6.68-fold higher signal than free DiR (Fig. 3K). Consistent with the fluorescence imaging results, HPLC quantification further demonstrated a pronounced spatial difference in GEM distribution after JTP implantation (Fig. S5, Supporting Information). The GEM accumulation was significantly higher in tumor tissue compared with blood and the opposite abdominal wall. These results demonstrate that upon implantation, JTP can be effectively retained within the surgical bed and sustainably release its loaded drug, enhancing intratumoral chemotherapeutic accumulation while minimizing peripheral distribution and systemic toxicity.

### 3.4 JTP presented precise CTC capture and excellent tumoricidal performance in vitro

**Fig. 4:**
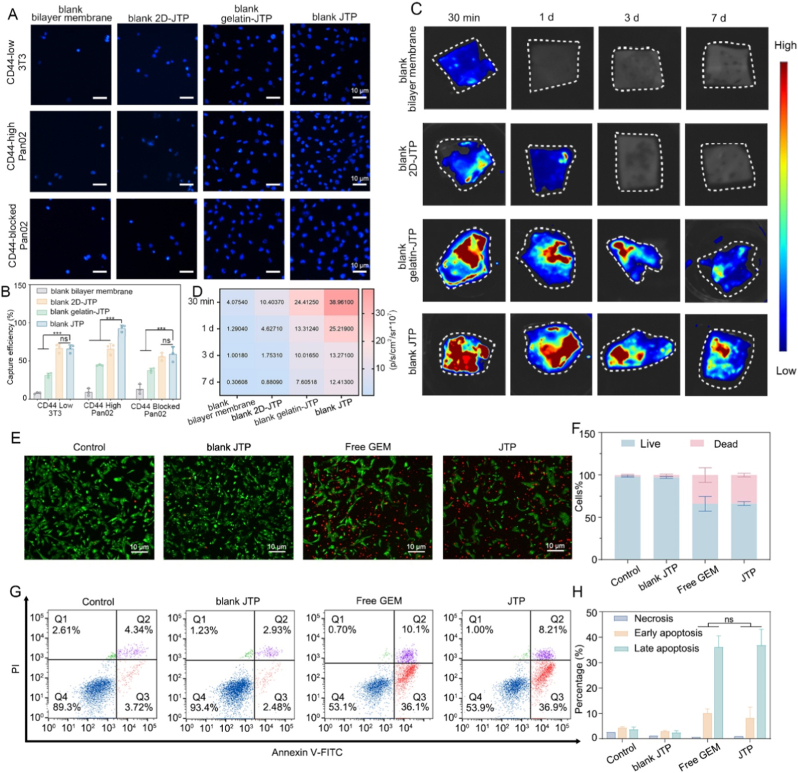
Cell capture performance and in vitro pharmacodynamic evaluation of JTP. (A) Fluorescence images of Hoechst-stained CD44-low 3T3, CD44-high Pan02 and CD44-blocked Pan02 cells incubated with different scaffolds in vitro. Scale bar = 10 μm. (B) Quantification of captured CD44-low 3T3, CD44-high Pan02 and CD44-blocked Pan02 cells on various scaffolds. (C) Bioluminescence imaging of Pan02-Luc cells captured on different scaffolds after implantation and retrieval from the surgical site of orthotopic PDAC-bearing mice. (D) Quantitative analysis of bioluminescence intensity on the retrieved scaffolds, reflecting the relative tumor cell capture efficiency of each material (n = 3). (E) Calcein-AM/PI staining of Pan02 cells treated with different scaffolds. Scale bar = 10 μm. (F) Quantification of live/dead cell ratios from Calcein-AM/PI staining (n = 3). (G) Annexin V/PI flow cytometry analysis of Pan02 cells after different treatments. (H) Quantitative analysis of apoptotic cell populations determined by Annexin V/PI staining (n = 3). Data were presented as mean ± S.D., ***P < 0.001, ns P > 0.05.

The intraoperatively implantable JTP comprised three functionally integrated layers: a PCL backing layer to prevent tissue adhesion, a GEM-loaded middle layer for unidirectional drug release, and a gelatin/HA inner sponge layer for rapid hemostasis and efficient CTC interception, thereby providing a coordinated intraoperative strategy for intraoperative tumor management. Considering its direct contact with surgical tissues during implantation, cytocompatibility was first assessed to ensure intraoperative biosafety. As shown in Fig. S6 (Supporting Information), NIH/3T3 fibroblast cells adhered and proliferated well on JTP and inner layer surfaces, confirming the favorable biocompatibility [[31], [32], [33]]. In contrast, the blank bilayer exhibited markedly reduced cell viability of 62.06% due to its hydrophobicity. Although hydrophobicity could minimize adhesion, it strictly hindered cell attachment and proliferation, resulting in poor biocompatibility. Importantly, the Janus-inspired architecture of JTP reconciles these conflicting interfacial requirements, featuring a hydrophilic and porous surface oriented toward the surgical cavity to promote cell compatibility and biological interaction, while maintaining an anti-adhesive hydrophobic layer toward the peritoneal side. This asymmetric interfacial design renders JTP particularly suitable for intraoperative implantation, where simultaneous biocompatibility at the resection interface and adhesion prevention on the non-target side are both essential. Western blotting pronounced CD44 expression in Pan02 cells, whereas negligible CD44 levels were detected in the non-tumor NIH/3T3 cells (Fig. S7, Supporting Information), confirming the HA-CD44 interaction as the molecular basis for tumor-specific capture. Accordingly, Pan02 and NIH/3T3 cells were employed as CD44-high tumor cells and CD44-low non-tumor controls while CD44-blocked Pan02 cells were served as control. For eliminating potential interference from GEM-induced cytotoxicity on tumor cell capture, a drug-free JTP was prepared to evaluate the intrinsic CTC capture capability both in vitro and in vivo. And, cell suspensions were incubated on the membrane for 3 h, followed by washing to remove unbound cells and Hoechst staining to visualize adherent ones. As shown in Fig. 4A and B, the blank bilayer membrane retained the fewest cells, which could be attributed to its dense and planar fibrous surface that hindered cell infiltration and physical trapping. The Janus-inspired membrane without 3D structure (blank 2D-JTP) slightly improved cell adhesion due to the incorporation of a hydrophilic gelatin/HA layer, yet its 2D configuration still limited effective capture. In contrast, trilayer membrane without HA (blank gelatin-JTP), benefiting from its 3D sponge-like porous network, markedly enhanced Pan02 cell capture, achieving 7.11-fold and 1.48-fold higher capture efficiencies than the bilayer membrane and 2D-JTP, respectively. These results indicate that a 3D porous architecture is critical for physical confinement of tumor cells, while biochemical recognition is required for capture specificity.

However, the absence of HA-mediated biochemical recognition resulted in comparable adhesion of NIH/3T3 cells, indicating insufficient selectivity of cell capture based on physical confinement alone. Notably, JTP combining porous confinement with HA-mediated recognition, exhibited the most efficient and selective capture, being 2.08 and 1.41 times higher than 2D-JTP and gelatin-JTP, respectively, while maintaining low attachment of NIH/3T3 cells. Moreover, blocking CD44 significantly reduced Pan02 cell capture on JTP and no longer significantly different from that on blank gelatin-JTP. Collectively, these findings confirm that the combination of a 3D sponge structure and HA-CD44 affinity enables efficient and tumor-specific capture, satisfying the functional demand for intraoperative CTC interception.

Encouraged by the in vitro capture specificity, we further evaluated the in vivo interception performance of JTP in an orthotopic postoperative PDAC model described above. After tumor removal, different membranes were implanted into the postoperative tumor cavity to capture disseminated luciferase-transfected Pan02 cells (Pan02-Luc cells), and the captured tumor cells were quantitatively evaluated based on bioluminescence intensity. As shown in Fig. 4C, both the bilayer membrane and trilayer membrane without 3D structure (2D-JTP) exhibited weak bioluminescent signals of Pan02-Luc cells, reflecting limited tumor cell capture. And, trilayer membrane without HA (gelatin-JTP) achieved transient enrichment within 30 min but lost retention over time, suggesting unstable physical trapping. In sharp contrast, the Janus-inspired membrane with 3D structure (blank JTP) showed markedly enhanced capture and durable retention of Pan02-Luc cells, with signal intensity 3.74- and 1.60-fold higher than 2D-JTP and gelatin-JTP at 30 min, respectively, and remained 1.63-fold stronger than gelatin-JTP after 7 days (Fig. 4D). The above results show that this 3D Janus-inspired membrane can quickly absorb blood and target CD44-positive tumor cells to achieve rapid and continuous CTC capture. The in vivo model used in this study has limitations. The combination of residual tumor and exogenously administered tumor cells does not fully recapitulate the complexity of clinical tumor dissemination and may amplify CTC-related processes. In this context, exogenous tumor cells were intentionally introduced to provide a controlled and reproducible CTC-like input, enabling quantitative evaluation of capture efficiency within a defined time window. Therefore, this model represents an accelerated and stringent scenario for functional validation.

To evaluate the local chemotherapeutic efficacy enabled by unidirectional GEM release, the in vitro cytotoxic performance of JTP was investigated. As shown in Fig. S8 (Supporting Information), the half maximal inhibitory concentration (IC50) of JTP against Pan02 cells was 38.23 μM, which was comparable to that of free GEM (36.22 μM). The results showed that JTP could release GEM to effectively kill tumor cells. Consistently, live/dead cell staining further confirmed that JTP induced tumor cell death at a level similar to free GEM (Fig. 4E and F), whereas the drug-free Janus-inspired membrane (blank JTP) exhibited negligible red fluorescence, demonstrating excellent cytocompatibility of the carrier scaffold. To further elucidate the underlying mechanism of JTP-induced tumor cell death, Annexin V/PI flow cytometric analysis was performed (Fig. 4G and H). Neither the untreated control nor the blank JTP group exhibited detectable apoptotic signals, confirming the non-cytotoxic nature of the Janus-inspired scaffold itself. On the contrary, both free GEM and JTP significantly increased the proportion of early and late apoptotic cells, while the number of necrotic cells was extremely small. This finding demonstrated that the GEM-loaded JTP primarily triggered programmed apoptotic pathways rather than passive necrosis. The above results show that JTP can effectively and continuously release GEM to induce tumor cell apoptosis. Collectively, these results demonstrate that JTP enables sustained local release of GEM, effectively inducing apoptotic tumor cell death while maintaining excellent carrier biocompatibility, thereby supporting its role as a localized.

### 3.5 JTP effectively prevented postoperative recurrence of PDAC via coordinated CTC interception and localized chemotherapy

**Fig. 5:**
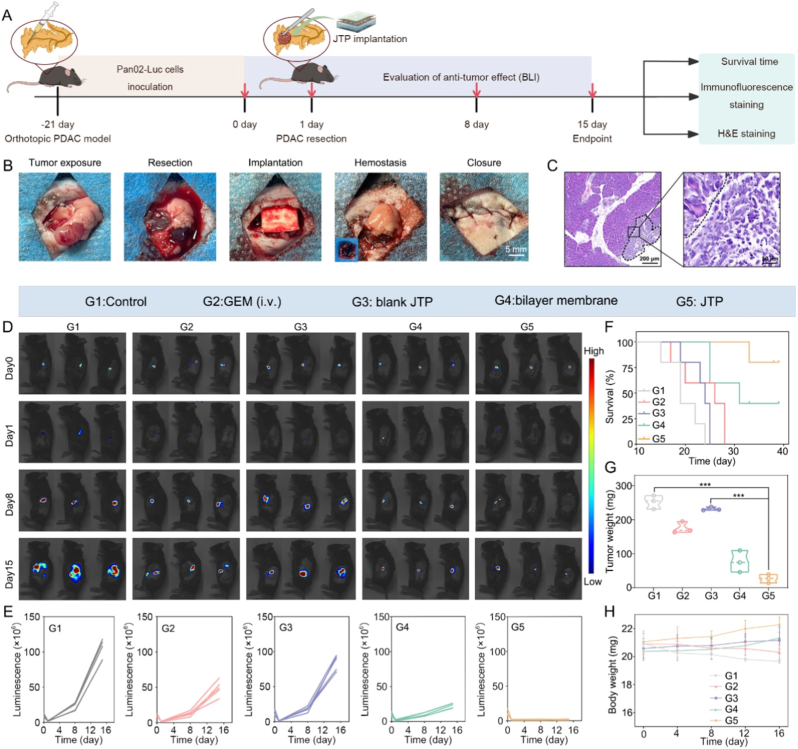
In vivo evaluation of different postoperative treatment strategies in PDAC tumor resection model. (A) Schematic illustration of the experimental design, including orthotopic implantation, surgical resection, and different treatments. (B) Intraoperative photographs showing tumor exposure, resection, membrane implantation, hemostasis, and closure. (C) H&E staining of residual tumor cells at the surgical margin. (D, E) In vivo bioluminescence imaging and quantitative analysis of tumor recurrence. (F) Kaplan-Meier survival curves of mice in different treatment groups (n = 5). (G) Tumor weight measurements at day 15 (n = 3). (H) Body weight of PDAC-bearing mice after different postoperative treatments (n = 8). Data were presented as mean ± S.D., ***P < 0.001, ns P > 0.05.

Given the clinical necessity to simultaneously control intraoperative bleeding, interrupt bleeding-driven tumor dissemination, JTP that integrates CTC interception and local GEM delivery held great promise for postoperative PDAC therapy, thereby contributing to more effective prevention of recurrence and metastasis. The incomplete PDAC tumor resection model was established via inoculating Pan02-Luc cells into the pancreatic tail of C57BL/6 mice, followed by surgical excision of visible tumor to mimic the clinical scenario of postoperative residual disease and recurrence (Fig. 5A). To replicate the clinical scenario of a tumor-positive incisal margin, tumor tissues were surgically excised under sterile conditions and subsequently subjected to different postoperative treatments, followed by surgical closure (Fig. 5B). The treatments included intravenous GEM (GEM i.v. group), drug-free patch implantation (blank JTP group, without drug loading), the bilayer membrane integrating GEM-loaded PLA and BL (Bilayer membrane group, drug-only group), and multifunctional Janus-inspired therapeutic patch implantation (JTP group), respectively.

The postoperative survival rate was more than 95%, which confirmed the reliability of the model construction. In Fig. 5C, Hematoxylin and eosin (H&E) staining showed different degrees of residual tumor cells in the cut edge and adjacent tissues, indicating that simple surgical resection failed to completely remove invasive PDAC, creating a potential risk of recurrence. Postoperative recurrence was monitored through in vivo bioluminescence imaging (Fig. 5D and E). In the control group and the blank JTP group, strong bioluminescence signals appeared after surgery, increasing by 47.57 times and 56.22 times, respectively, in 14 days. Compared with the control group, intravenous injection of GEM showed a brief inhibitory effect in the early stage. However, the recovery of bioluminescence intensity was observed in a short time due to the limited accumulation of GEM in the tumor. In contrast, the recurrence time of the bilayer membrane group was longer than that of the intravenous GEM group, but the fluorescent signal gradually increased over time, indicating that the positioning of controlled-release GEM alone was not enough to inhibit recurrence. The bioluminescence intensity of the JTP group increased slightly, indicating that it has the strongest inhibitory effect on tumor recurrence. Its remarkable efficacy was attributed not only to the release of GEM, but also to the synergistic effect of membrane hemostasis and tumor cell capture characteristics.

Owing to the combination of hemostasis and GEM-induced residual tumor cells killing, JTP significantly prolonged the survival time with an 80% survival rate on day 38 (Fig. 5F). In addition, JTP reduced tumor weight from 252.2 mg (Control) to 27.0 mg on day 15, accounting for only 35.33% of the bilayer membrane group (Fig. 5G). Notably, no significant body weight loss was observed throughout the treatment, indicating a favorable safety profile (Fig. 5H). H&E staining of the heart, liver, spleen, lung, or kidney across all treated groups from healthy C57BL/6 mice revealed no evident morphological abnormalities (Fig. S9, Supporting Information). Besides, serum biochemical assays and hematological tests revealed no significant differences between the JTP and control group (Figs. S10 and S11, Supporting Information). Collectively, these findings demonstrate that the biodegradable JTP can sustain therapeutic drug concentrations at the tumor site over prolonged periods while maintaining minimal systemic toxicity.

### 3.6 JTP suppressed hepatic metastasis

Encouraged by the robust antitumor efficacy of JTP, its ability to suppress distant metastasis was further evaluated. Given the high propensity of PDAC for hepatic metastasis, livers were excised from orthotopic PDAC-bearing mice for ex vivo fluorescence imaging, gross observation, and histological analysis [34,35].

**Fig. 6:**
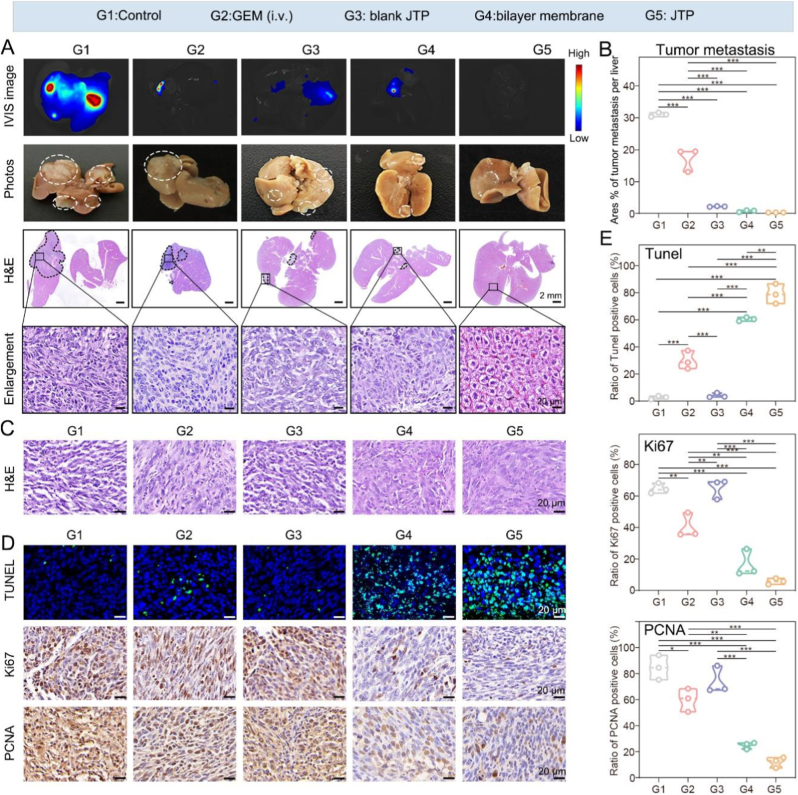
Resistance to tumor recurrence and metastasis after treatment by JTP. (A) IVIS images, photo images and H&E section images of liver metastasis after tumor recovery in different treatment groups. (B) Percentage of metastatic area to total hepatic cross-sectional area in different treatment groups (n = 3). (C) Representative H&E staining of primary tumor sections showing cellular density, nuclear morphology, and tissue architecture. Scale bar = 20 μm. (D) Representative TUNEL staining and Ki67 and PCNA immunostaining of tumor sections indicate apoptosis and proliferation, respectively. Scale bar = 20 μm. (E) Quantification of TUNEL-positive, Ki67-positive, and PCNA-positive cells. (n = 3). Data were presented as mean ± S.D., ***P < 0.001, **P < 0.01, *P < 0.05.

As shown in vivo images in Fig. 6A, liver in the control group displayed intense fluorescence signals, indicating that without timely postoperative intervention, PDAC readily developed significant hepatic metastasis. In contrast, in the group implanted with blank JTP, hepatic fluorescence intensity was markedly reduced, demonstrating that the CTC-capturing capabilities of the JTP may contribute to reduced PDAC liver metastasis by limiting tumor cell dissemination. And, nearly undetectable hepatic fluorescence in the JTP-treated group compared with the control and GEM (i.v.) groups, indicating that JTP not only released GEM in a sustained manner but also actively captured residual circulating tumor cells at the surgical site, thereby significantly reducing the metastatic burden. Moreover, as shown in (Figs. S12 and S13, Supporting Information), quantification of CTCs demonstrated that JTP implantation markedly reduced the number of CTCs in peripheral blood throughout the postoperative period, while the time-dependent enhancement of tumor cell killing observed on the JTP scaffold further confirmed that the captured CTCs were not only retained locally but were progressively eliminated through sustained GEM release, thereby continuously reducing the viable metastatic cell population at the postoperative site. Consistently, numerous metastatic nodules formed by migrated tumor cells were observed in the control group, whereas the GEM-treated group exhibited visibly fewer but still detectable metastatic foci, indicating that systemic administration of GEM alone was insufficient to effectively prevent PDAC liver metastasis. In contrast, photographic images of the isolated livers showed substantially fewer and smaller metastatic nodules following JTP treatment. H&E staining further confirmed these observations, displaying notably diminished metastatic foci after JTP treatment. Quantitative analysis of the percentage of metastatic area to total hepatic cross-sectional area demonstrated a pronounced decrease in the JTP-treated mice, which was 1.14% of the control mice (Fig. 6B), underscoring the potent inhibitory effect on postoperative liver metastasis. Notably, blank JTP groups and bilayer membrane groups exhibited moderate inhibition of hepatic metastasis compared with untreated mice, confirming that tumor capturing and localized chemotherapy provide partial protection against tumor dissemination, while neither group achieved complete suppression of metastatic lesions. These results indicate that JTP patch efficiently inhibits tumor metastasis through its tumor trapping and localized chemotherapeutic functions. Negligible macroscopic or microscopic metastatic nodules were detected in other major organs in the JTP-treated group, indicating an effective inhibition of secondary tumor dissemination. These findings demonstrate that the localized and sustained drug release achieved by the JTP effectively prevents postoperative tumor metastasis, highlighting its superior therapeutic advantage in postoperative pancreatic cancer management.

As shown in Fig. 6C–H&E staining showed that tumors in the control and blank JTP groups exhibited dense cellularity and typical malignant morphology with enlarged nuclei and disorganized architecture. Intravenous injection of free GEM only caused limited nuclear shration and fragmentation, indicating that its cytotoxicity was relatively weak. Local implantation of bilayer membrane could effectively increase the concentration of focal drugs and improve the ability to kill tumors. In sharp contrast, the JTP group exhibited the most pronounced tumor destruction with widespread cellular lysis and tissue disruption, consistent with the Janus-inspired membrane's integrated design, where rapid hemostasis, capture of residual tumor cells and circulating tumor cells, and localized GEM delivery collectively enhanced tumor clearance and prevented local dissemination. In addition, the JTP group showed a strong TUNEL signal, with an apoptosis number of 79.14%, which was significantly higher than that of the free GEM group (30.09%) and the bilayer membrane group (60.60%). In contrast, only a small number of TUNEL-positive cells were observed in the control group and the blank JTP group. Immunostaining for Ki67 and PCNA revealed substantial suppression of proliferation in the JTP group, with Ki67-positive cells reduced to 5.91% compared with 64.66% in the control and PCNA-positive cells reduced to 12.22% compared with 84.88% in the control (Fig. 6D and E). These results demonstrate that the JTP simultaneously induces tumor apoptosis and inhibits proliferation through the combined effects of rapid hemostasis, CTCs capture, and unidirectional intratumoral chemotherapy. This multifunctional platform provides sustained suppression of postoperative tumor recurrence while minimizing systemic exposure.

## 4 Conclusion

In summary, we introduced an effective intraoperative intervention strategy for bridging surgery and localized chemotherapy for postoperative PDAC management. We designed a multifunctional Janus-inspired therapeutic patch (JTP) as an intraoperatively implantable platform that unifies rapid hemostasis, interception of bleeding-driven circulating tumor cells, and localized chemotherapy within a single material system. By integrating a hydrophobic anti-adhesive backing, a unidirectional gemcitabine-releasing nanofibrous layer, and a bioactive 3D gelatin/hyaluronic acid sponge at the surgical interface, JTP enables coordinated regulation of the postoperative tumor microenvironment directly at the resection site. Compared with conventional single-layer systems, which often suffer from functional coupling between hemostasis and drug delivery, the present multilayer design enables spatial separation of these functions. Hydrogel-based systems typically lack spatial control over drug distribution, resulting in non-specific diffusion, while bilayer configurations provide only partial functional separation. In contrast, the Janus-inspired multilayer architecture allows hierarchical organization of hemostasis, tumor cell interception, and localized chemotherapy, thereby improving functional coordination within the postoperative microenvironment.

Several intraoperative adjuvant strategies, including intraoperative radiotherapy (IORT) and hyperthermic intraperitoneal chemotherapy (HIPEC), have been introduced into clinical practice to improve local tumor control after surgical resection. However, their adoption in routine clinical practice remains limited by strict patient selection, the need for specialized facilities and multidisciplinary expertise, and potential treatment-related toxicities. In contrast, JTP offers a more broadly applicable material-based intraoperative strategy by integrating rapid hemostasis, tumor cell interception, and localized chemotherapy within a single implantable platform.

This rational Janus-inspired design transforms the surgical wound from a passive trauma site into an active therapeutic interface, achieving rapid hemostasis, effective tumor cell interception, and sustained local chemotherapeutic exposure. Moreover, JTP markedly suppressed postoperative tumor recurrence and hepatic metastasis, significantly prolonging survival in an orthotopic PDAC resection model, while maintaining favorable biosafety. Collectively, this study establishes JTP as a promising intraoperative strategy that bridges surgical resection and localized chemotherapy. Beyond PDAC, the proposed Janus-inspired therapeutic concept provides a generalizable framework for intraoperative control of tumor dissemination and recurrence, offering new opportunities for surgical oncology interventions at the material-tissue interface.

## Funding

The authors gratefully acknowledge the Young Talents' Research Ability Enhancement Program of the First Affiliated Hospital of Wannan Medical College; the project on integrated development of 10.13039/501100014980Wannan Medical College (XQHR202421); the Chief Expert Project of Clinical Medical Transformation in Anhui Province (202427b10020032); the Major Natural Science Project of Universities in Anhui Province (2023AH040254), and Wuhu Science and Technology Program Project (2024kj043).

## CRediT authorship contribution statement

**Xuan Pan:** Conceptualization, Data curation, Formal analysis, Investigation, Methodology, Project administration, Visualization, Writing – original draft, Writing – review & editing. **Xiaoming Wang:** Data curation, Funding acquisition, Supervision, Visualization, Writing – review & editing. **Qin Dang:** Project administration, Resources, Software, Writing – original draft. **Longjiang Chen:** Validation, Visualization. **Guannan Wang:** Resources, Software. **Xu Wang:** Data curation, Formal analysis. **Shihang Xi:** Formal analysis, Investigation. **Huaqing Zhang:** Conceptualization, Project administration, Supervision, Visualization, Writing – review & editing. **Xiaosan Fang:** Supervision, Writing – original draft, Writing – review & editing.

## Declaration of competing interest

The authors declare that they have no known competing financial interests or personal relationships that could have appeared to influence the work reported in this paper.

## Data Availability

Data will be made available on request.

## References

[1] Stoop T.F., Javed A.A., Oba A., Koerkamp B.G., Seufferlein T., Wilmink J.W., Besselink M.G. (2025). Pancreatic cancer. Lancet.

[2] Zhao Y., Zheng Y., Zhu Y., Ding K., Zhou M., Liu T. (2023). Co-delivery of gemcitabine and triapine by calcium carbonate nanoparticles against chemoresistant pancreatic cancer. Int. J. Pharm..

[3] Rajesh C., Cummings R.D., Radhakrishnan P. (2025). Unraveling the glyco-immunity nexus in pancreatic cancer. Mol. Cancer.

[4] Zhao R., Xiao Q., Wu Y., Zhang W., Liu J., Zeng Y., Zhan J., Cai Y., Fang C. (2024). Dual-crosslinking immunostimulatory hydrogel synchronously suppresses pancreatic fistula and pancreatic cancer relapse post-resection. Biomaterials.

[5] Kotb A., Hafeji Z., Jesry F., Lintern N., Pathak S., Smith A.M., Lutchman K.R.D., de Bruin D.M., Hurks R., Heger M., Khaled Y.S. (2024). Intra-operative tumour detection and staging in pancreatic cancer surgery: an integrative review of current standards and future directions. Cancers (Basel).

[6] Pizon M., Zimon D., Pachmann U.A., Pachmann K. (2015). Tumorspheres cultured from circulating epithelial tumor cells (CETCs) in pancreatic cancer patients having cancer stem cells properties and resistance to standard chemotherapeutics. J. Clin. Oncol..

[7] Kayahara M., Nagakawa T., Ueno K., Ohta T., Takeda T., Miyazaki I. (1993). An evaluation of radical resection for pancreatic cancer based on the mode of recurrence as determined by autopsy and diagnostic imaging. Cancer.

[8] Manabe T., Okino H., Maeyama R., Mizumoto K., Nagai E., Tanaka M., Matsuda T. (2004). Novel strategic therapeutic approaches for prevention of local recurrence of pancreatic cancer after resection: trans-tissue, sustained local drug-delivery systems. J. Contr. Release.

[9] Cornejo G., Blumenfeld P., Hubert A. (2024). Intra-operative radiation for pancreatic cancer: initial experience at the hadassah medical center. J. Clin. Oncol..

[10] Tohme S., Simmons R.L., Tsung A. (2017). Surgery for cancer: a trigger for metastases. Cancer Res..

[11] Zhang Z., Kuang G., Zong S., Liu S., Xiao H., Chen X., Zhou D., Huang Y. (2018). Sandwich-like fibers/sponge composite combining chemotherapy and hemostasis for efficient postoperative prevention of tumor recurrence and metastasis. Adv. Mater..

[12] Castro B.G.R., Dos Reis R., Cintra G.F., Sousa M.M.A., Vieira M.A., Andrade C. (2018). Predictive factors for surgical morbidities and adjuvant chemotherapy delay for advanced ovarian cancer patients treated by primary debulking surgery or interval debulking surgery. Int. J. Gynecol. Cancer.

[13] Fransgaard T., Caspar Thygesen L., Gögenur I. (2021). The impact of postoperative complications and delay of adjuvant chemotherapy on oncological outcomes in patients with colorectal cancer. Colorectal Dis..

[14] Huang X., Zhang G., Tang T.-Y., Gao X., Liang T.-B. (2022). Personalized pancreatic cancer therapy: from the perspective of mRNA vaccine. Milit. Med. Res..

[15] Ren B., Cui M., Yang G., Wang H., Feng M., You L., Zhao Y. (2018). Tumor microenvironment participates in metastasis of pancreatic cancer. Mol. Cancer.

[16] Xiao Y., Fan Y., Tu W., Ning Y., Zhu M., Liu Y., Shi X. (2021). Multifunctional PLGA microfibrous rings enable MR imaging-guided tumor chemotherapy and metastasis inhibition through prevention of circulating tumor cell shedding. Nano Today.

[17] Wang S., Cao M., Chen Y., Lin J., Li J., Wu X., Dai Z., Pan Y., Liu X., Liu X., Lin L.T., Wu J., Liu J., Zhong Q., Yuan Z. (2025). Advancements and applications in radiopharmaceutical therapy. Chin. J. Nat. Med..

[18] Lee J., Kim E., Kim K.-J., Rhie J.W., Joo K.I., Cha H.J. (2024). Protective topical dual-sided nanofibrous hemostatic dressing using mussel and silk proteins with multifunctionality of hemostasis and anti-bacterial infiltration. Small.

[19] Zhong Z., Gan L., Feng Z., Wang W., Pan X., Wu C., Huang Y. (2024). Hydrogel local drug delivery systems for postsurgical management of tumors: Status quo and perspectives. Mater. Today Bio.

[20] Xu L., Bai E., Zhu Y., Qin J., Du X., Huang H. (2023). pH-Responsive hydrogel as a potential oral delivery system of baicalin for prolonging gastroprotective activity. Pharmaceutics.

[21] Wang M., Zeng X., Wuang X., Zhang Z., Guo S., Deng Y., Li X., Yao L., Li J., Wong L., Bai W., Feng X. (2025). A biodegradable antimicrobial oligomer-containing hydrogel for drug-resistant bacteria-infected skin wound treatment. Pharm. Sci. Adv..

[22] Zhao Y., Liu Y., Liu Z., Ren K., Jiao D., Ren J., Wu P., Li X., Wang Z., Han X. (2024). In situ nanofiber patch boosts postoperative hepatocellular carcinoma immune activation by trimodal combination therapy. ACS Nano.

[23] Ma Y., Li M., Fu X., Li D., Gao C., Li Z., Zhang Y., Gao Z., Geng H., Li G., Ni S., Cui J. (2025). Engineering of nanofibers embedded with targeted nanoparticles breaks redox levers for glioblastoma therapy. Adv. Funct. Mater..

[24] Cao K., Zhou Y., Shen Y., Wang Y., Huang H., Zhu H. (2024). Combined photothermal therapy and cancer immunotherapy by immunogenic hollow mesoporous silicon-shelled gold nanorods. J. Pharmaceut. Sci..

[25] Tam S.W., Cheung A.K.L., Qin P., Zhang S., Huang Z., Yung K.K.L. (2025). Extracellular silica nanomatrices promote in vitro maturation of anti-tumor dendritic cells via activation of focal adhesion kinase. Adv. Mater..

[26] Wolinsky J.B., Colson Y.L., Grinstaff M.W. (2012). Local drug delivery strategies for cancer treatment: gels, nanoparticles, polymeric films, rods, and wafers. J. Contr. Release.

[27] Goyal R., Macri L.K., Kaplan H.M., Kohn J. (2016). Nanoparticles and nanofibers for topical drug delivery. J. Contr. Release.

[28] Khan A., Le Pivert M., Ranjbari A., Dragoe D., Bahena-Uribe D., Colbeau-Justin C., Herrero C., Rutkowska-Zbik D., Deschamps J., Remita H. (2025). Cu-Based MOF/TiO2 composite nanomaterials for photocatalytic hydrogen generation and the role of copper. Adv. Funct. Mater..

[29] Cao W.T., Ma C., Mao D.S., Zhang J., Ma M.G., Chen F. (2019). MXene‐Reinforced cellulose nanofibril inks for 3D‐Printed smart fibres and textiles. Adv. Funct. Mater..

[30] Lv Y., Xu Y., Zhang S., Xie S., Wang B., Sun T., Zhang X., Yao S., Zhang H., Wang L. (2025). Rapidly photocurable and strongly adhesive hydrogel‐based sealant with good procoagulant activity for lethal hemorrhage control. Adv. Funct. Mater..

[31] Stadlmayr S., Mautner A., Bacher M., Peter K., Mentler A., Schulz S., Lichtenegger H., Brecker L., Bismarck A., Naghilou A. (2025). Holistic analysis of material properties in phylogenetically diverse spider silks and their influence on cell adhesion. Adv. Funct. Mater..

[32] Awashra M., Jokinen V. (2025). Superhydrophobic cell-repellent microstructures: plastron-mediated inhibition of A549 epithelial cell adhesion. Small.

[33] Smita S.S., Pramanik K. (2025). Development of silk Fibroin/Gelatin/PCL tri-polymeric complex nanofibrous two-dimensional mat for epithelial tissue regeneration. Chem. Eng. J..

[34] Liu Q., Zhang R., Michalski C.W., Liu B., Liao Q., Kleeff J. (2020). Surgery for synchronous and metachronous single-organ metastasis of pancreatic cancer: a SEER database analysis and systematic literature review. Sci. Rep..

[35] Tanaka M., Mihaljevic A.L., Probst P., Heckler M., Klaiber U., Heger U., Büchler M.W., Hackert T. (2019). Meta-analysis of recurrence pattern after resection for pancreatic cancer. BJS.

